# Prevalence of Refractive Errors Among Medical Students of Raichur Institute of Medical Sciences, Raichur, Karnataka, India

**DOI:** 10.7759/cureus.58915

**Published:** 2024-04-24

**Authors:** Karishma Munoli, Siddesh Harpanalli, Sandhya Holkar, Soumya M Malkhed, Bylappanavara Girish, Koli R Vannura

**Affiliations:** 1 Department of Ophthalmology, Raichur Institute of Medical Sciences, Raichur, IND

**Keywords:** hyperopia, astigmatism, refractive error, myopia, hypermetropia

## Abstract

Introduction

Refractive errors (REs) are on the rise among medical students due to the high academic pressure of long hours of reading and their association with close technology. Uncorrected REs cause impaired vision and blindness with considerable morbidity. Documenting the prevalence of REs among medical students helps with early detection and appropriate remedial measures to prevent eye morbidity.

Aim

The aim of the study was to find the prevalence of REs among medical students at Raichur Institute of Medical Sciences, a medical institution in Raichur City, Karnataka, India.

Methodology

This hospital-based cross-sectional study was conducted with a total of 425 medical students. An examination of the eye for REs was carried out using a streak retinoscope. The REs were noted in myopia <-0.5 diopters (D), hypermetropia >+0.5 D, and astigmatism >0.5 cylinder D. The data was statistically subjected. Categorical measurements have been presented as frequency (percentage). The chi-square test was applied to the association between the parameters. A p-value less than 0.05 was considered statistically significant.

Results

Among the 425 observed participants, 160 (37.6%) subjects had REs. Among the REs in the total population, myopia 78 (18.4%) was the most prevalent, followed by hypermetropia and astigmatism, both with 41 (9.6%) and 41 (9.6%) prevalence, respectively. Gender-wise and age-wise, myopia was highly prevalent in both genders and in all age groups except the 20 years age group.

Conclusion

The prevalence of REs in our study among medical students is a matter of concern, although it is less compared to other previous studies in different geographical areas of India. Regular checkups, early detection, and immediate treatment are very important to prevent further ocular complications.

## Introduction

Refractive error (RE) is a condition in which the optical system of the non-accommodating eyes is not able to bring parallel rays of light to focus on the retina [1]. Uncorrected RE is the second cause of global visual impairment, next to cataracts [2]. REs are the second major cause of blindness in India after cataracts. Over a quarter of outpatient attendance at eye clinics and hospitals is due to REs [3]. Uncorrected REs created a greater impact on learning and academic success [4]. The prevalence of REs was high among medical students, and they were unaware of it [5]. For the prevention of blindness, WHO and the International Agency for the Prevention of Blindness identified uncorrected REs as one of the main priority programs launched through the Vision 2020 initiative to eradicate blindness [6]. Uncorrected RE has been responsible for half of all visual impairment worldwide, affecting over 124 million people [7]. The literature indicated that TV watching from a short distance, using electronic devices for a long time, using dim light while reading, family history, gender (female), and near-work activity are major causes of REs [8-10]. These medical professionals spend long hours reading, and this group is in close contact with technology [5].

Detection of the REs at an early age assumes greater attention for corrective measures and also to improve the academics and lifestyle of the medical students. There is no such study undertaken in the Raichur district of Karnataka in southern India. Hence, this study was conducted among medical students of Raichur Institute of Medical Sciences, Raichur district of southern India.

## Materials and methods

Study setting, study population, and study period

The institutional-based cross-sectional study was carried out among medical students of Raichur Institute of Medical Sciences, a government medical institution in Karnataka state, southern India. The institution has an intake of 150 students in each academic year. Overall, in five academic years, approximately 750 students have been officially enrolled in the institution during our study period. The study was conducted from July 19, 2023 to March 19, 2024. The selected study group, which met inclusion criteria, included all five academic years’ total of 425 medical students of both sexes, aged 18-25 years, who participated in the study. Subjects with a past history of ocular surgery, trauma, spasm of accommodation, congenital anterior segment abnormalities, asthenopic complaints, dropped-out students, not present during the study period, and unwilling participants were excluded from the study.

Sampling procedure

The pilot study was conducted on 50 convenient samples; the prevalence was found to be 36%, and based on this pilot study, the final sample was calculated.

The formula used to calculate the final sample size was as follows [11]: N = Z21-α/2*P*(1-P)/d2. Here, N represents the number, P denotes prevalence, α signifies the type 1 error, d indicates absolute precision, and Z stands for 1.96 at 95%.

After applying the above formula for the pilot study prevalence of 36%, the final sample obtained was 355.

Ethical consideration

Ethical permission was obtained from the institutional ethical committee prior to the study (RIMS/IEC/2023-24/43; date: July 18, 2023). The selected study population had been explained about the study’s objectives, and written consent was obtained that mentioned the purpose of the study, methods, risks, and benefits. The study subjects were given a free choice to withdraw from the study at any given point in time without citing any reasons. Each study participant was allotted a unique study number to mask their identity during the study process. The confidentiality of the study population data has been maintained. The overall data was used only for study purposes.

Operational definition and examination of eyes for REs

Definitions used for the analysis were the following: myopia was defined as spherical equivalent (SE) less than minus 0.5 diopters (D), hypermetropia was defined as SE more than 0.5 D, and astigmatism was defined as a cylinder of less than minus 0.5 D [12]. A total of 712 medical students present during our study period were offered to fill out the pretested questionnaire after informed consent. The questionnaire contained demographic data (age, gender, and year of study) and past ocular history (surgery, trauma, spasm of accommodation, congenital anterior segment abnormalities, and authentic complaints). After analyzing the questionnaire, the 425 participants with no past history of ocular problems were selected and included as the study group, and they underwent ocular examination.

The examination of the eyes to detect REs was as follows: streak retinoscopy was used to measure spherical refraction (hypermetropia and myopia) and cylindrical refraction (astigmatism). For the relaxation of the study subject accommodation, both eyes were kept open for relaxation of accommodation, and they were asked to focus on a far target or dilate the eyes with a cycloplegic agent (one drop of 1% cyclopentolate hydrochloride was used as a cycloplegic agent in our study). Maintenance of a calm environment and providing dimmed light were essential for better contrast of the papillary reflex. The examiner was seated at arm’s length from the study subject, and the study subject had sat uprightly. For better visualization of the red reflex, the streak retinoscope light was directed at the study subject’s right pupil. Horizontally across the study subject’s pupil, the retinoscope had been swept. With the movement of the retinoscope, the comparison of the movement of the reflex in the pupil was done (the reflex moving in the same direction as the retinoscope streak). The streak of the retinoscope had been rotated to the horizontal position, and it had been swept across the pupil vertically. The streak retinoscope had been rotated, and the sweeping for the oblique meridians was at 45 degrees and 135 degrees. The REs (spherical or astigmatic) had been determined. For spherical errors, the observed reflex had consistent direction, brightness, speed, and width in all meridians. For astigmatic errors, the observed reflex appeared different in different meridians [13,14].

The spherical component had been determined by achieving neutrality with the correct lenses. This was accomplished by holding different lenses in front of the examined eye. The neutral point was achieved when the examiner swept across the pupil, and the reflex filled the entire pupil with no perceived movement. The neutral point was double-checked with the observation of the reflex movement when moving slightly backward and forward from the normal working distance [14,15].

A repetition of the examination for the study subject’s left eye had been done. The examiner looked through the retinoscope using his or her left eye and held the instrument in his or her left hand.

Interpretation of REs

To determine the final spherical error, the operator calculated the number of diopters that needed to be offset based on the working distance; it is done by subtracting the inverse of the working distance, in meters from the retinoscope. If the working distance between the retinoscope and lens was 100 cm, then 1/1m (1.0 diopters) would be subtracted from the retinoscope to get the spherical error. The sphere-cylindrical corrections that neutralized the RE, minus the working distance corrections, were the recording results. The first number indicated spherical power in diopters; plus power represented hypermetropia, and minus power was depicted as myopia. The second and third numbers indicated astigmatism; the second number was the power of the cylinder, and the third number indicated the axis on which the cylinder was neutralized [16].

The same instrument and calibrated ophthalmologist examined all study participants.

Data analysis

The data were entered and analyzed with IBM SPSS Statistics for Windows, Version 25.0 (Released 2022; IBM Corp., Armonk, NY, USA). The collected data was subjected to statistical analysis. Statistical analysis was also performed using IBM SPSS Statistics for Windows, Version 25.0. Results from categorical measurements are presented as frequency (percentage). The association between the parameters was done using the chi-square test. A p-value less than 0.05 was considered statistically significant.

## Results

The prevalence of REs was found to be 160 (37.6%) in our study subjects. Among REs, myopia was found in 78 (18.6%), followed by hypermetropia in 41 (9.6%), and astigmatism in 41 (9.6%). In a total of 175 male study population, 84 (48%) had REs; among them, myopia (41, 23.4%) was the highest, followed by hypermetropia (24, 13.7%) and astigmatism (19, 10.9%). In a total of 250 female population, 76 (30.4%) study subjects had REs; among them, myopia was found in 37 (14.8%) study subjects, followed by astigmatism in 22 (8.8%) population and hypermetropia in 17 (6.8%) participants. A statistically significant difference was observed between males and females for myopia and hypermetropia (p < 0.05) (Table 1).

**Table 1:** REs and gender * Statistically significant RE, refractive error

RE	Total (N = 425), N (%)	Male (N = 175), N (%)	Female (N = 250), N (%)	p-value
Myopia	78 (18.4)	41 (23.4)	37 (14.8)	0.030*
Hypermetropia	41 (9.6)	24 (13.7)	17 (6.8)	0.017*
Astigmatism	41 (9.6)	19 (10.9)	22 (8.8)	0.48
Total	160 (37.6)	84 (48)	76 (30.4)	

In the comparative evaluation of REs among age groups of 18, 19, 20, 21, 22, and 23 years, myopia was most prevalent in all age groups except the 20 years age group. With three different REs associated with age groups, it was found that a significant association was observed between hypermetropia and age (p < 0.008), and in overall REs among all age groups, there was a significant association with age and REs (p < 0037). In gender and age comparisons, it was found that hypermetropia in all age groups in the male population was significantly associated (p < 0.001), and in overall REs of the male population in all age groups, there was significantly associated (p < 0.020) (Table 2).

**Table 2:** REs, gender, and age * Statistically significant RE, refractive error

RE	Gender	Age (years)							p-value
		18 (N = 66)	19 (N = 89)	20 (N = 59)	21 (N = 72)	22 (N = 89)	23 (N = 50)	Total (N = 425)	
Myopia	Female	4 (6.1)	8 (9.0)	4 (6.8)	12 (16.7)	5 (5.6)	4 (8.0)	37 (8.7)	0.552
	Male	2 (3.0)	10 (11.2)	4 (6.8)	4 (5.6)	13 (14.6)	8 (16.0)	41 (9.6)	0.276
	Total	6 (9.1)	18 (20.2)	8 (13.6)	16 (22.2)	18 (20.2)	12 (24.0)	78 (18.4)	0.23
Hypermetropia	Female	2 (3.0)	2 (2.2)	4 (6.8)	5 (6.9)	2 (2.2)	2 (4.0)	17 (4.0)	0.847
	Male	3 (4.5)	2 (2.2)	9 (15.3)	3 (4.2)	3 (3.4)	4 (8.0)	24 (5.6)	0.001*
	Total	5 (7.6)	4 (4.5)	13 (22)	8 (11.1)	5 (5.6)	6 (12.0)	41 (9.6)	0.008*
Astigmatism	Female	3 (4.5)	8 (9.0)	3 (5.1)	3 (4.2)	2 (2.2)	3 (6.0)	22 (5.2)	0.491
	Male	3 (4.5)	1 (1.1)	0	3 (4.2)	8 (9.0)	4 (8.0)	19 (4.5)	0.167
	Total	6 (9.1)	9 (10.1)	3 (5.1)	6 (8.3)	10 (11.2)	7 (14.0)	41 (9.6)	0.709
Total	Female	9 (13.6)	18 (20.2)	11 (18.6)	20 (27.8)	9 (10.1)	9 (18.0)	76 (17.9)	0.152
	Male	8 (12.1)	13 (14.6)	13 (22.0)	10 (13.9)	24 (27.0)	16 (32.0)	84 (19.8)	0.020*
	Total	17 (25.8)	31 (34.8)	24 (40.7)	30 (41.7)	33 (37.1)	25 (50.0)	160 (37.6)	0.037*

## Discussion

The overall prevalence of REs in our study after observing 425 samples was 160 (37.6%), which is a matter of concern because the study population consists of medical students who are vulnerable to REs as per the previous studies. The prevalence rate in our study was only 37.6% compared to other studies in different parts of India, which have found more than 50% prevalence; studies conducted in different regions by Garg et al. and Kathrotia et al. in India have reported the REs at 54% and 54.95%, respectively [17,18]. The high level of REs among medical students is due to the long and exhaustive study schedule [19].

In our study, myopia was commonly occurring in RE compared to hypermetropia and astigmatism in the overall study population and also in both genders. Various studies have also reported the highest prevalence of myopia compared to other REs [17,18,20]. The high prevalence of myopia among medical students could be attributed to the high educational workload and intensive study regimen with prolonged near work [21,22]. Recent theory indicates that prolonged near work causes blurred retinal images and leads to myopia. Blurred retinal images stimulate biochemical structural changes in the sclera and choroid that cause axial elongation [23]. The National Program for Control of Blindness and Visual Impairment (NPCB and VI) was started in 1976 by the central government of India to achieve the goal of reducing blindness to 0.3% by 2020. The surveys done in 2001-2002, 2006-2007, and 2015 showed a prevalence of blindness of 1%, and in 2015, it was 0.45%. Cataracts and RE are major causes of blindness, so stressing early detection and correction is very important to prevent blindness [24].

Our survey is important because it reported data about the prevalence on a regional basis and specific population groups (medical students). Medical students are at high risk because of excess reading pressure to cover a lot of medical topics and the high use of technology in day-to-day life. Our reported data about the prevalence of REs in this geographic area assumes greater national importance to focus attention on the government to implement the programs of reduction of blindness across the length and breadth of the country, and our data will also help researchers in other parts of the country study the prevalence of REs among medical students in their localities.

Our study has been conducted in this geographical area; the results cannot be generalized to other areas. The study used a streak retinoscope to measure the REs; in rare instances, it might have produced astigmatic errors if it was off the axis, and if the instrument operated at an incorrect working distance, it would result in spherical errors.

## Conclusions

The prevalence of REs in our study is a great concern, and myopia was found to have the highest prevalence compared to other REs. Uncorrected REs are the main cause of irreversible ocular damage and blindness. Gathering data on region-specific prevalence is necessary for national policy decisions to launch health programs on a broad scale without regional disparity.

Regular eye checkups, early detection, and immediate treatment are very important to prevent further ocular complications. Medical students, due to their excess academic pressure, are more vulnerable to REs, so examination of these groups is very important to detect the REs at an early stage and to take remedial measures to prevent permanent ocular damage.
